# Type 2 transglutaminase in the nucleus: the new epigenetic face of a cytoplasmic enzyme

**DOI:** 10.1007/s00018-023-04698-8

**Published:** 2023-01-25

**Authors:** Federica Rossin, Fabiola Ciccosanti, Manuela D’Eletto, Luca Occhigrossi, Gian Maria Fimia, Mauro Piacentini

**Affiliations:** 1 Department of Biology, University of Rome ‘Tor Vergata’, Via Della Ricerca Scientifica 1, 00133 Rome, Italy; 2 Department of Epidemiology, Preclinical Research and Advanced Diagnostics, National Institute for Infectious Diseases IRCCS ‘L. Spallanzani’, Rome, Italy; 3 Department of Molecular Medicine, University of Rome “La Sapienza”, Rome, Italy

**Keywords:** Transcriptional factors, Epigenetic, Interactome, TG2, cBAF

## Abstract

One of the major mysteries in science is how it is possible to pack the cellular chromatin with a total length of over 1 m, into a small sphere with a diameter of 5 mm “the nucleus”, and even more difficult to envisage how to make it functional. Although we know that compaction is achieved through the histones, however, the DNA needs to be accessible to the transcription machinery and this is allowed thanks to a variety of very complex epigenetic mechanisms. Either DNA (methylation) or post-translational modifications of histone proteins (acetylation, methylation, ubiquitination and sumoylation) play a crucial role in chromatin remodelling and consequently on gene expression. Recently the serotonylation and dopaminylation of the histone 3, catalyzed by the Transglutaminase type 2 (TG2), has been reported. These novel post-translational modifications catalyzed by a predominantly cytoplasmic enzyme opens a new avenue for future investigations on the enzyme function itself and for the possibility that other biological amines, substrate of TG2, can influence the genome regulation under peculiar cellular conditions. In this review we analyzed the nuclear TG2’s biology by discussing both its post-translational modification of various transcription factors and the implications of its epigenetic new face. Finally, we will focus on the potential impact of these events in human diseases.

## Transglutaminase type 2

Transglutaminases, also referred to as protein–glutamine g-glutamyltransferases (EC 2.3.2.13), are a family of 8 active and 1 inactive (Band 3 of red blood cells) enzymes present in the human genome that catalyze the acyl-transfer reaction between glutamine and primary amines [15, 39]. Transglutaminase type 2 (TG2) is a peculiar multifunctional ubiquitously expressed member of the TG family that catalyzes post-translational modifications of proteins through both Ca^2+^ dependent and independent reactions. Several unique features, including its ubiquitous expression, widespread localization, binding to and hydrolysis of guanine nucleotides, distinguish TG2 from the other transglutaminases. TG2 changes from a closed to an opened form, under increased intracellular Ca^2+^ concentrations, exerting cross-linking and transamidase activities. Moreover, in addition to its cross−linking activity, TG2 might also act as a GTP−binding protein that mediates intracellular signalling by coupling the alpha−1 beta−adrenergic receptor to the phospholipase C−gamma1. Under physiological circumstances the enzyme may also act as protein disulphide isomerase (PDI) [13, 19]. A serine/threonine kinase activity for TG2 has been also reported in vitro. This potential kinase activity is able to phosphorylation the p53 tumor suppressor protein [46], histones H1–4 [47] and retinoblastoma (Rb) protein [45]. More recently due to the improvement of the omics approaches an important function of TG2 as scaffold protein has been reported under different physio/pathological settings [26, 50, 52, 71, 73]. The 3D structure of TG2 is composed of four domains: an NH2-terminal β-sandwich domain; a catalytic core domain containing a catalytic triad for the acyl-transfer reaction (Cys277, His335 and Asp358); a β-barrel1 domain, containing GDP/GTP-interacting residues, that is involved in receptor signalling and a β-barrel2 domain [25]. In the last few years, it has been established that TG2 switches its 3D structure from a nucleotide-bound “closed” to the transamidation/PDI prone “open” conformation and these substantial 3D changes influence the interaction with multiple substrates and/or binding partners that are essential to carry out its biological functions [59, 77]. In fact, in low intracellular nM Ca^2+^ concentrations, it mainly acts as a GTPase-supporting growth. By contrast, when cells are injured and the intracellular Ca^2+^ concentration increases, it acts as a crosslinking, playing a key role in autophagy, or PDI enzyme and if the cellular damage is irreversible inducing cell death [13, 56, 62]. In keeping with these findings TG2 has been shown to be involved in the pathogenesis of the major human diseases [50, 52, 75]. Another TG2 feature is its intracellular distribution, in fact, although it is predominantly found in cytoplasm it is also distributed in various subcellular locations including plasma membrane, mitochondria and nucleus [2, 4, 57].

## The nuclear TG2

**Fig. 1:**
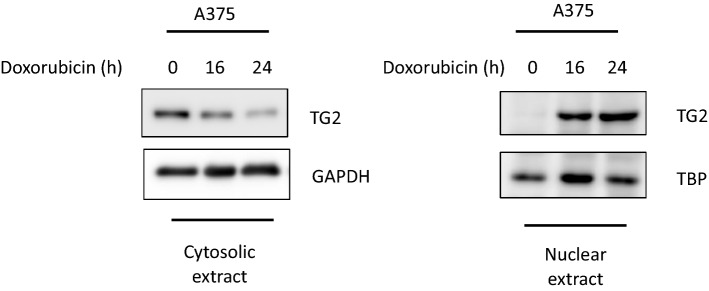
Western blot analysis showing cytosolic (left) and nuclear (right) protein expression of TG2 in A375 melanoma cells treated with doxorubicin for 16 and 24 h. GAPDH and TBP were used as loading control for cytosolic and nuclear fraction, respectively

Although the first consistent report claiming the presence of TG2 in the nucleus was published more than 40 years ago [9], little attention has been dedicated to the enzyme intracellular localization and to its potential epigenetic regulatory role in the nucleus. In fact, only after 15 years from the initial claim, the Ballestar group [6], using monodansylcadaverine (DNC) as donor amine, indicated that core histones can act as glutaminyl substrates for the TG2-catalyzed reaction. In particular, the authors showed that out of the 18 glutamines present in the four histones (namely, glutamine 95 of H2B; glutamines 5, 19, and 125 of H3; glutamines 27 and 93 of H4; and glutamines 24, 104, and 112 of H2A) can play a role acting as amine acceptors in transamidating reactions catalyzed by TG2 [6]. The same year we confirmed, using a proteomic approach, that the histone 2b can act as transglutaminase type 2 substrate in dying cells [60]. A few years later, Kim group confirmed that the core histones can act as TG2 substrates in senescent fibroblasts [31]. TG2 dependent modification of histones correlate with its presence at the chromatin level. Indeed, it has been reported that TG2 becomes strongly associated with the chromatin during neutrophils differentiation changing the expression of key molecules involved in chemotaxis, phagocytic capacity and superoxide production [5, 12]. Another study, revealed the presence of the enzyme as being mostly associated with euchromatin, confirming the notion that nuclear TG2 especially impacts on the regulation of gene expression via post-translational modification and/or interaction with transcriptional factors [58]. A very important contribution to the complex intracellular behaviour of TG2 has been achieved by the yeast two-hybrid approach showing that the enzyme can interact with the importin-a3, thus confirming the possibility for the TG2 to shuttle between the cytoplasmic vs the nuclear environment [57]. Accordingly, the primary sequence of TG2 contains two putative nuclear localization signals (NLS) located at positions 259–263 (DILRR) and 597–602 (PKQKRK); however, bioinformatic studies indicated that the PKQKRK residues (597–602) can be predicted to be the functional NLS [57]. Moreover, a putative leucine-rich nuclear export sequence (NES) at the position ^657^LHMGLHKL^664^, near the C terminus, has been also identified [69]. The nuclear translocation of the enzyme as well as its activation is observed under peculiar stressful cellular conditions, such as those reported in Fig. 1 [37, 85]. In keeping with this, TG2 can translocate into the nucleus in hepatocytes isolated from wild-type mice treated with ethanol but not in TG2 knock-out ones, promoting the release of mitochondrial cytochrome C with a consequent differential modulation of apoptosis [70].

## Direct and indirect TG2-dependent regulation of transcriptional factors

**Fig. 2:**
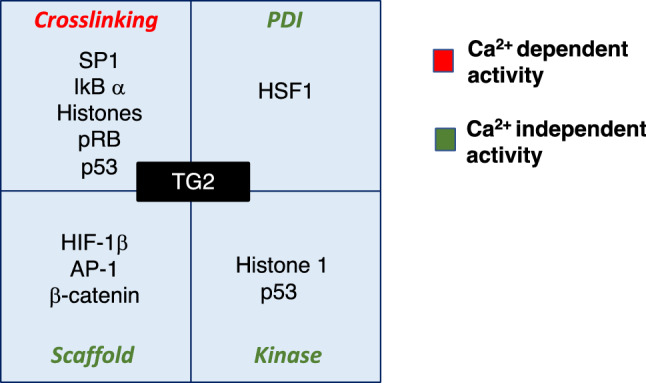
Representation of the transcriptional factors modified by TG2 and the enzymatic activities involved

In the last 25 year several groups have shown that the nuclear TG2 can catalyse different post-translational modifications (acting as cross-linking, PDI and kinase) of important transcription factors thus playing a direct key regulatory role on gene transcription (Fig. 2). In addition, the enzyme modifies the status of inhibitory/stimulatory proteins, that act as rheostats regulating the access to the nucleus of other transcriptional factors, thus indirectly interfering with their transcriptional activity.

### pRB

Retinoblastoma protein (pRB) was the first tumor suppressor discovered and years of research have revealed that is a master regulator of biological pathways influencing cell fate, such as cell growth, cell-cycle, differentiation, senescence, self-renewal, replication, genomic stability and apoptosis. It has been shown that, the retinoblastoma gene product is post-translationally modified by a TG2-catalyzed reaction in the early stages of human leukemic monocyte lymphoma cells (U937 cells) undergoing apoptosis [54]. In fact, apoptosis of U937 cells is characterized by the rapid disappearance of the 105,000–110,000-molecular-weight pRB forms concomitantly with the appearance of a smear of immunoreactive products with a molecular weight greater than 250,000. The Rb protein regulates cell growth/death fate depending on its phosphorylation state, mainly acting via its interaction with E2F1 [82]. Interestingly, the nuclear TG2 has been shown to modulate the function of Rb protein in different ways. In fact, in U937 the TG2 activity leads to the Rb protein polymerization and consequently to the degradation of E2F1 which has as final outcome the cell growth arrest/apoptosis. By contrast, in other cell lines the presence of nuclear TG2 is associated with the Rb protein stabilization protecting it from degradation by caspase-7 and as outcome an anti-apoptotic action [8]. These observations indicate that in general, nuclear TG2 polymerizes Rb protein under high Ca^2+^ concentrations through its transamidating activity. However, the outcome differs depending on cell types and treatments, suggesting that intranuclear Ca^2+^ concentrations could be important in determining these TG2 opposing functions.

### Sp1

Sp1, a glutamine-rich protein belonging to the Sp-family of transcription factors, plays central roles in the regulation of a number of genes that influence cell survival and proliferation. Kojima and co-workers were the first who reported that in liver, consequently to the ethanol induced apoptosis, there is an enhanced nuclear accumulation of TG2 leading to the cross-linking inactivation of Sp1 followed by the reduced expression of the Sp1-dependent gene, c-Met. The comparison of the TG2 + / + vs mice TG2−/− mice showed markedly reduced hepatocyte apoptosis and Sp1 cross-linking following ethanol or anti-Fas treatment [73]. A further study evidenced the TG2 dependent crosslinking and inactivation of Sp1, here resulting in reduced expression of epidermal growth factor receptor and cell death [76]. An upregulation of Sp1 transcriptional activity, dependent on the crosslinking action of nuclear TG2 was also reported [21]. Therefore, the crosslinking and inhibition of Sp1 by TG2 may be a feedback mechanism to control the excessive expression of TG2, since the transcriptional activation of the TG2 gene, TGM2, is mediated also by Sp1 [65, 74].

### HSF1

We have recently demonstrated that TG2 regulates cellular proteostasis and cell's adaptation to stress by acting as a PDI [63]. At the molecular level our results indicated that the TG2 is responsible for the activation of the heat shock factor 1 (HSF1), the main transcriptional factor regulating the inducible heat shock response. Under physiological condition, HSF1 is present in the cytoplasm as inactive monomer in association with Hsp90 and Hsp70. Under cellular stressful conditions condition, HSF1 is released by these HSPs and subsequently moves into the nucleus binding the Heat Shock Elements (HSEs) on the promoters of the target genes. The HSF1 activation requires the transition from the monomeric to the trimeric form of the transcription factor. Specifically, two cysteine residues (cys35 and 105), localized in the DBD domain, are essential for the formation of disulphide bridges in the trimeric HSF1 [1]. Interestingly we found that HSF1 trimerization is mediated by the PDI activity of TG2. In fact, mice lacking TG2 display a markedly impaired response to the HS due to the absence of TG2-dependent HSF1 trimerization. These findings have also been confirmed in human models, where the TG2 inhibition affects HSF1 activation [63]. Whole RNA-seq analysis in wild-type vs TG2 knockout mouse MEFs confirmed that the enzyme has a profound effect on gene expression, since cells lacking TG2 display a high number of upregulated or downregulated genes. Interestingly, Gene Set Enrichment Analysis highlighted many HS genes downregulated in cells lacking TG2, thus confirming that the enzyme is necessary for a proper response to the HS [61]. Overall, these studies indicate that the TG2 nuclear translocation can be an important element in the regulation of gene expression particularly under cellular stressful conditions.

### Wnt

It has been recently shown that TG2 influences the overall cellular transcriptome profile and specifically the Wnt signaling. Indeed, TG2 is necessary for a proper induction of the Wnt pathway, that results strongly downregulated when the enzyme is absent [61]. Mechanistic studies revealed that nuclear TG2 interactome includes several proteins known to be involved in the regulation of the Wnt signaling and specifically TG2, acting as a scaffold protein, physically interacts with β-catenin. Other studies correlate TG2 to β-catenin activation, indeed, it has been shown that TG2 inhibition with ZDON suppressed canonical Wnt signaling through a reduction of β-catenin thus preventing osteoarthritis [22]. Moreover, in ovarian cells TG2 physically associates with and recruits c-Src, which in turn phosphorylates β-catenin, releasing it from E-cadherin and rendering it available for transcriptional regulation [11]. The TG2-dependent regulation of the Wnt pathway has been shown to play a key role also in the differentiation of preadipocytes to lipid storing adipocytes [49].

Interestingly, recent evidence showed a crosstalk between the Wnt and HSF1 pathways [18, 86]. In this context, we recently found that TG2 could cooperate with HSF1 to regulate Wnt signaling. In fact, key components of the Wnt/β-catenin pathway are also downregulated in cells lacking HSF1, thus suggesting that TG2 regulates HSF1 and this axis could control Wnt [50, 52, 61].

### NF-kB

About 20 years ago some reports highlighted the tight relationship existing between TG2 expression and NF-kB, the major regulator of genes involved in inflammatory and immune responses. Interestingly, both the genetic and the pharmacological inhibition of TG2 resulted in a reduced NF-kB/DNA interaction [32]. An important finding was reported by Lee et al. showing that TG2 activates NF-kB through a novel molecular mechanism. The cross-linking of the IkBα by TG2 limits the availability of this molecule for the interaction and the consequent inhibition of NF-kB highlighting a non-conventional mechanism by which TG2 can activate NF-kB [33]. The TG2-dependent regulation of NF-kB is evolutionary conserved. In *Drosophila*, transglutaminase (TG) crosslinks the nuclear factor-κB (NF-κB)-like transcription factor Relish active N-terminal fragment Relish-N to inhibit its nuclear translocation and the consequent regulation of many genes. It is interesting to note that the putative Relish-N TG-modified Gln residues are located in the DNA-binding region of Relish-N [41]. From these studies it is possible to conclude that TG2 has a complex regulatory role on NF-kB that is stimulus-dependent and may have an opposite outcome.

### p53 family

P53 is a multifunctional transcription factor that suppresses tumor growth through regulation of dozens of target genes with diverse biological functions. Its activity is inhibited in the vast majority of human tumors, either by gene mutations or overexpression of the p53 repressor MDM2.

There is a general consensus that TG2 expression is upregulated in various tumors and this is associated with p53 instability [23, 27, 42, 79]. In renal cell carcinoma it has been shown that the downregulation of TG2 stabilizes p53 expression, thereby inducing an increase in apoptosis. TG2 in a crosslinking independent manner promotes autophagy-dependent p53 degradation by binding to p53 and p62 and driving them inside the assembling autophagosomes. Deletion mutants of p62, p53 and TG2 revealed that the PB1 and the transactivation domain of p62 directly interacts, respectively, with the β-barrel and the N terminus domains of TG2. Interestingly, TG2 acts as an effective chaperone molecule for p53 transportation into the autophagosomes trough p62 without the involvement of its catalytic activities. However, when inside the autophagosome TG2 is activated by an increase in calcium concentration leading to the crosslinks of p53 [27]. At the functional level the increase in p53 stability due to TG2 inhibition synergizes with the administration of a DNA-damaging anti-cancer drug such as doxorubicin inducing apoptosis in RCC cell lines and determining a reduced tumor volume in a xenograft model [28].

Interestingly it has also been reported that p53 is a substrate for a putative serine/threonine kinase activity of TG2 at p53 Ser(15) and Ser(20) residues that are critically important for the interaction of p53 with Mdm2 [46]. However, these in vitro studies have not yet found a validation in in vivo settings.

Increased TG2 promoted angiogenesis by inducing p53 degradation, leading to the activation of HIF-1α. Indeed, the TG2 dependent p53 depletion increased the availability of p300 for HIF-1α-p300 binding inducing angiogenesis [34].

Interestingly, it has also been reported that ΔNp63α, another member of the p53 family, can be stabilized by TG2 leading to maintenance of cancer stem cell features [20].

## The epigenetic TG2 regulatory face

Post-translational modifications of histone are key epigenetic regulatory features that have important roles in the regulation of chromatin structure and in the activation and repression of gene transcription. The enzymes responsible for these epigenetic marks are often referred to as writers, erasers and readers. Writers introduce various chemical modifications on DNA and histones, erasers remove these chemical tags and readers identify and interpret those modifications [7]. These histone modifications also provide binding platforms for diverse transcription factors, such as chromatin remodels, histone chaperones, DNA/histone-modifying enzymes and general transcription factors [24].

Many findings reported that TG2 is able to trigger different modifications of histones, making possible to suggest its role as a “writer”. More than two decades ago Shimizu et al. described the finding that an acyl transfer reaction, catalyzed by the nuclear TG2, could lead to the crosslinks of H2B and H4 histones in starfish sperm [66, 67]. Accordingly, it was confirmed that all four mammalian core histones (H2A, H2B, H3 and H4) may act as glutaminyl substrates for TG2-catalyzed reaction and their crosslinking contributes to chromatin condensation associated with the induction of apoptosis [6]. A very recent study suggests that the TG2 transamidating activity on chromatin is regulated by its substrate accessibility rather than by primary sequence determinants or by the existence of pre-existing post-translational modifications [40].

These potential epigenetic features of the enzyme have been neglected for many years. However, recently Farrelly et al. convincingly demonstrated that the serotonin (also known as 5-hydroxytryptamine; 5-HT), an excitatory neurotransmitter, can be covalently attached to histone H3 by TG2. In this study it was shown that the glutamine 5 of the histone 3 (H3Q5) acts as the binding site for the serotonylation by TG2. The authors highlighted that H3Q5 serotonylation can occur in parallel with the trimethylation of the H3 on lysine 4 (H3K4me3), which has been shown to play an important role on gene transcription, by recruiting the TFIID and consequently the RNA polymerase II to the chromatin [17]. In addition, they identified genomic loci that precisely display differential regulation of H3K4me3Q5ser during differentiation, they also found that several thousands of protein-coding genes display H3K4me3Q5ser enrichment mostly within gene promoters. However, future studies should clarify when and in which cells this TG2-dependent tuning of gene expression plays a key regulatory role. In this regard, a recent study evidenced that the presence of the H3Q5ser post-translational modification potentiates H3K4me3 function by either stabilizing H3K4me3 or enhancing its interaction with downstream effectors and thereby suggesting a molecular mechanism for gene expression regulation [87]. In keeping with this, it is interesting to note that this TG2-dependent modification of H3K4me3Q5ser has been detected not only in brain, but also in other tissues (heart, and testicular tissues and peripheral blood mononuclear cells).

Other evidence about TG2 dependent modification of histones have been recently reported from the Maze’s group demonstrating that the TG2-catalyzed dopaminylation of histone 3 is associated with aberrant expression of addiction- and synaptic plasticity-related neuronal genes in response to cocaine consumption [36].

### TG2 nuclear interactome

A whole comparative transcriptomic analysis using TG2 knock out fibroblasts vs wild type confirmed a marked effect of TG2 on gene expression modulation [61]. In particular, this study highlighted the enzyme’s transcriptional regulation of the expression of many chaperones (heat shock proteins and several BAG family members), Wnt family members as well as RNAs encoding for many neural regulatory proteins [61]. This TG2-dependent transcriptional regulatory action opens a new research area about the possible influence of neurotransmitters, whose level is dependent from mood/stress, on epigenome and consequently cell homeostasis. At the same time, it is important to recall that the TG2 catalytic activity can lead to the incorporation of many other biogenic amines (di-and polyamines, histamine) into histones opening an avenue for future studies linking epigenetic to cellular metabolism.

Another unexpected emerging aspect about the chromatin/remodelling role of TG2 is that related to the regulation of the SWI/SNF (SWItch/Sucrose Non-Fermentable) complex. In fact, it has been shown that TG2 physically interacts with the BAF250a (also known as ARID1a) protein which is a key component of the SWI/SNF complex (cBAF). In this case, TG2 catalyzes the covalent incorporation of polyamines into BAF250a [30]. cBAF is a key ATP-dependent chromatin remodelling complex playing an important tumour suppressor role. In mammalian, 3 versions of the BAF complex have been characterized on the basis of the presence of the different components (encoded by 29 genes); the BRG1/BRM-associated factor complex (cBAF), the polybromo containing complex (pBAF) and a non-canonical version of the complex (ncBAF) [48]. It is important to note that each functional eukaryote BAF complexes comprises a group of proteins (more than 10) that are able to remodel the way DNA is packaged. In fact, by its ATPase activity the complex can destabilize the histone–DNA interactions in the nucleosomes, this determines an extensive nucleosome rearrangement allowing the positive/negative fine-tuning of genes [3].

**Fig. 3:**
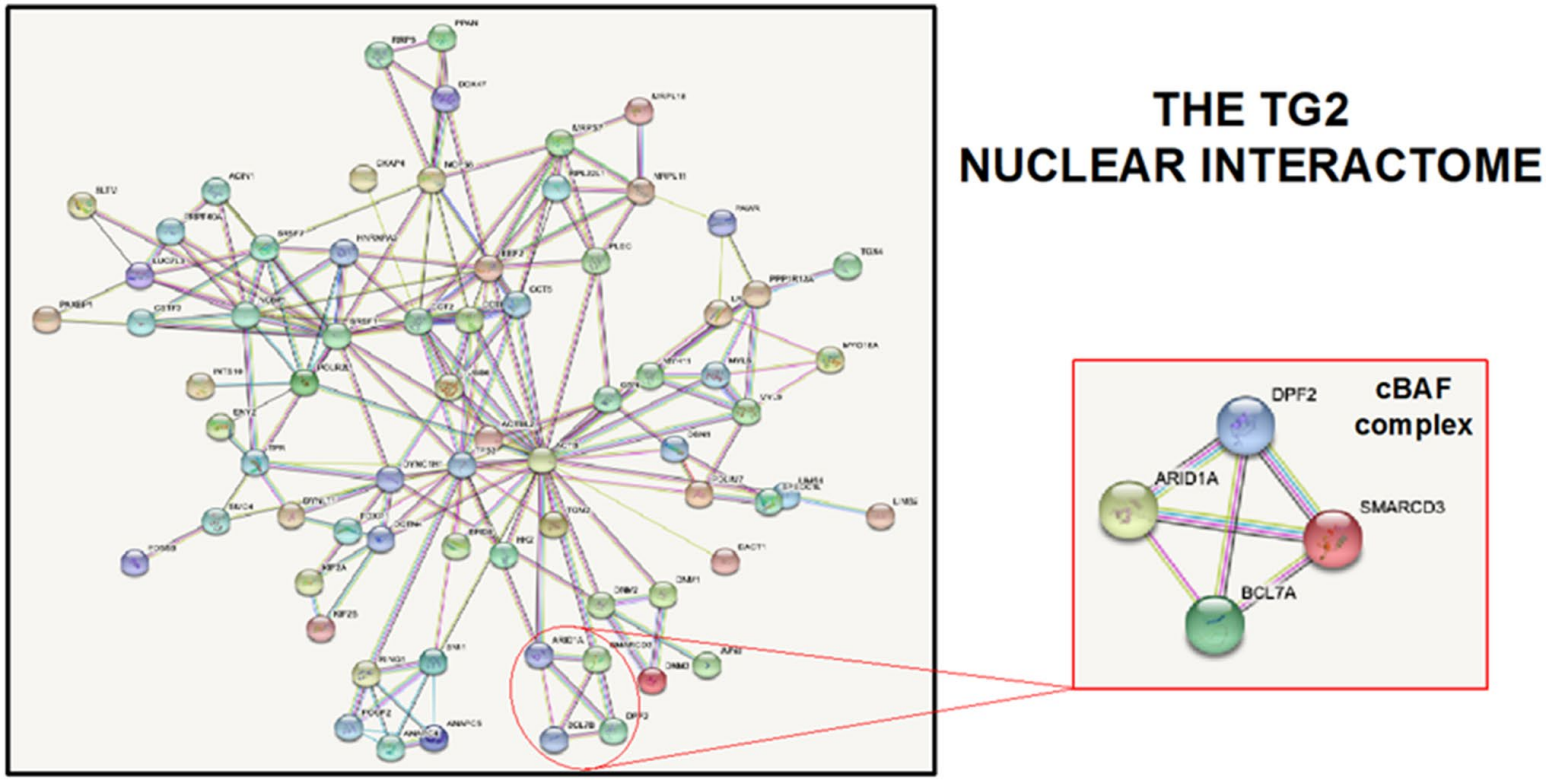
Protein–protein interaction network of the identified proteins into the nucleus of MEF cells. The network containing identified proteins was mapped using the STRING system (http://string-db.org/) based on evidence with different types. cBAF complex is showed in the inset

**Fig. 4:**
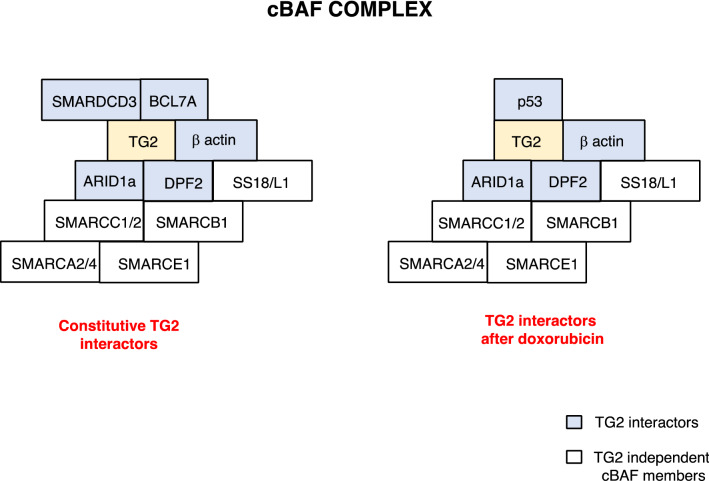
Representation of cBAF complex proteins interacting with TG2 into the nucleus before and after doxorubicin treatment

We have recently carried out the nuclear interactome of TG2 before and after doxorubicin treatment (Fig. 3). Our data confirm the interaction of TG2 with ARID1 and highlighted the enzyme binding with other four constitutive components of the cBAF complex (Fig. 4). Interestingly, some of these interactions are lost after treatment with doxorubicin which determines a TG2-dependent rearrangement of the complex including the recruitment of p53. This TG2-dependent interaction of p53 with the cBAF complex upon treatment with doxorubicin is particularly appealing due to the complex role of the cBAF complex in cancer. In fact, doxorubicin intercalates with DNA and inhibits the topoisomerase II, preventing the double helix from being released, consequently the DNA damage response and transcriptome are deregulated. For these reasons, doxorubicin is used alone or in combination with other chemotherapeutic agents in many cancer treatments. It has been reported that the tetramerization domain of p53 interacts with BAF60a/SMARCD3, but not with other components of the cBAF complex. It is interesting to note that the p53-dependent anti-tumor function is inhibited (apoptosis and cell cycle arrest) when its interaction with the BAF60a complex is disrupted by genetic approaches [53]. These findings suggest a tumor suppressing mechanism for the cBAF complex mediated by p53 activation. In line with this hypothesis, a recent report suggested a mechanism by which TG2 might be involved in DNA damage repair through its direct interaction with TOPOIIα [35]. On the basis of these evidence, it is likely that the TG2/TOPII action might require the co-operation of the newly assembled cBAF/p53 complex, observed after doxorubicin treatment.

### Potential TG2/cBAf interplay in disease

So far, no mutations associated with TG2 have been described in mammals and extensive exome sequencing data have not uncovered individuals with homozygous loss-of-function variants for TGM2 gene [78]. This seems to indicate that even heterozygous mutations must be lethal during early embryogenesis. However, in clear contrast with this assumption is the fact that TG2 knock out mice are viable, reproduce normally and do not have any obvious phenotypic abnormality. However, these mice display a large number of problems under stressful conditions compared to wild type animals [16, 43, 61]. In keeping with this, TG2 has been shown to play a role in the major human disorders including cancer, neurodegeneration, various forms of fibrosis as well as infectious and metabolic diseases [14]. This complexity could indicate that the enzyme can act as a co-factor in different settings according to its expression level, tissue and intracellular localization. To verify whether the epigenetic action of TG2 might help to unravel its biological function in the next paragraph we have analyzed the pathologies that share the involvement of TG2 and its interacting partners in the SWI/SNF complex.

The BAF complexes are generally considered as a transcriptional activator and antagonizes the Polycomb Repressor Complexes in the modulation of gene expression [81]. Nonetheless, repressing activity for the SWI/SNF has also been reported. BAF components are frequently perturbed in tumors. In particular, ARID1A loss in cancer cells leads to increased cell proliferation, migration, and invasion, as well as reduced cell apoptosis and chemosensitivity. However, the role of ARID1A in cancer is still not fully defined, in fact, some authors suggested a tumor suppression role, while others indicate its oncogenic function. For instance, a recent study performed on hepatocellular line HepG2 uncovered that ARID1A activates and represses roughly equal numbers of genes [55]. The genomic analysis of human ES cells revealed that depletion of ARID1A increases nucleosome occupancy repressing on a set of H3K4me3 associated promoters. Considering the serotonylation of this histone by TG2, this finding appears particularly interesting for the understanding its possible cooperation with ARID1a.

TG2 has been shown to play a role in hepatocarcinogenesis and when absent there is a significant up-regulation of the TRIB3 mRNA expression [61]. TRIB3 is a protein overexpressed in advanced grade HCC tissues and is closely correlated with poor prognosis. Interestingly, co-immunoprecipitation assays demonstrated that TRIB3 interacts with SMARCD3 in the nucleus, suggesting that it may regulate TRIB3 in HCCs [80].

It has been shown that ARID1 A mutations are very frequent in ovarian cancers. They are mainly frame-shift or nonsense mutations determining loss of ARID1A expression and correlates with disease late-stages. Interestingly, BCL7A is also expressed at low levels in ovarian cancer tissues and is correlated with shorter survival status. Matei and co-workers have shown in a number of studies the important role played by TG2 in this tumor. In particular they have shown that TG2, by activating the NF-kB, inhibits apoptosis induced by cisplatin and modulates the epithelial-to-mesenchymal transition (EMT), thus contributing to increased ovarian cancer invasiveness and metastatic potentials [84]. In line with these findings our interactome analysis has evidenced that p53 interacts with TG2 only after doxorubicin treatment and this can lead, as shown by Kim group, to its degradation via autophagy [27, 28], thus promoting the survival of cancer cells. Future studies should dissect out what is the functional meaning of the interaction of ARID1, BCl7a and TG2 in cancer cells.

It has been very recently reported that the BCL7A-containing SWI/SNF/BAF complex regulates the Wnt signaling and the mitochondrial bioenergetics [83]. Interestingly, TG2 is also involved in the modulation of the same pathways [61]. Indeed, in a cellular model of Huntington disease, TG2 inhibition de-repressed two key regulators of mitochondrial function, PGC-1α and cytochrome *c* [44, 64].

The chromatin-remodelling BAF or SWI/SNF complexes have been reported to be involved in the induction of a subset of IFN-inducible genes [38]. However, it is not completely defined whether the BAF complexes are needed for the cellular antiviral activities. A study reported that the knockdown of DPF2 protein in cells infected with influenza virus resulted either in decreased expression of RNA and viral proteins or increased expression of IFN-β and phosphorylation of STAT1 [68]. DPF2 functions as an adaptor protein that links the noncanonical NF-κB complex (RelB/p52) with the SWI/SNF chromatin-remodelling complex [72]. Interestingly in a recent study, we have shown that TG2 negatively regulates cGAs/STING/TBK1/IRF3 signaling pathway. In the absence of TG2, we found an increase in the IFN-β production and the downstream JAK/STAT pathway activation. In keeping with these findings, we observed an increase in the IFNβ production in bronchoalveolar lavage fluids from COVID-19-positive dead patients paralleled by a dramatic decrease of the TG2 expression in the lung pneumocytes [50, 52]. In keeping with these findings, newly we showed that, upon doxorubicin treatment (Fig. 1), TG2 translocates into the nucleus and catalyzes the formation of covalent cross-linked IRF3 (Interferon regulatory factor 3) dimers, thereby limiting the production of IFNβ in dying melanoma cells [51].

Finally, it has been shown that the hypoxia-inducible factor-1 alpha (HIF-1α) is directly regulated by the SWI/SNF chromatin-remodeling complex Indeed, the SWI/SNF components associate with the HIF-1α promoter resulting in a pronounced change in HIF-1α expression and consequently in its ability to transactivate target genes including TG2 [29, 64].

## Discussion

It is becoming clear that the full understanding of the biological role of TG2 must encompasses its nuclear localization. The TG2 nuclear function can be exploited in many ways through its transamidating activity leading to the incorporation of primary amines into the histones as well as modifying transcriptional factors either by crosslinking or amines incorporation. The transamidating activity requires increased level of calcium which is released by the intracellular stores (ER mitochondria) mainly under stressful cellular conditions. However, TG2 can also acts as PDI as in the case of HSF1 trimerization or as scaffold protein without the calcium requirement. The demonstration of the post-translational incorporation of neurotransmitters in the H3K4me3 opens unexpected scenarios for the so far unclear comprehension of the involvement of TG2 in many biological processes, but more in general for the possibility that the enzyme can influence gene expression under peculiar cellular events leading to metabolic reprogramming. For instances the serotonylation of proteins has been shown to participate in several processes as neurotransmission, insulin secretion and vascular muscle contractility.

TG2 catalyzed reactions may also lead to the covalent incorporation of other primary amines, such as putrescine, spermidine, spermine, histamine and noradrenaline. In this regard, it is well-documented the drastic increase of di- and polyamines observed in cancer cells [10]. This metabolic change can result in a dual effect on TG2 either by inhibiting its transamidating activity in the cytosol or leading to the incorporation of di- and polyamines in the histones in the nucleus.

An unexplored feature of TG2 biochemical activity, that can also play an important role in the chromatin post-translational modification, is its action as deamidating enzyme of specific glutamines. This TG2 activity plays a key role in celiac disease in which the deaminated gliadin acts as trigger for the production of pathogenetic antibodies specific for deamidated gluten peptides. Future studies should address as to whether the enzyme can also use this additional activity to modify the histones.

Finally, it is important to define the nature of the interaction of TG2 with the BAF complex components that we have reported in this review. In particular, it is the protein acting as a scaffold under peculiar cellular circumstances or are its BAF interactors undergoing TG2-catalyzed posttranslational modifications? The elucubrations reported in this review led to a number of open questions that need to be addressed; however, it highlights the function of TG2 in the epigenetic field and suggests additional view in the regulation of chromatin post-translational modifications. The comprehension of these novel functions may significantly contribute to our understanding of TG2 complex function and to clarify its involvement in diseases.

## Data Availability

No data availability.
